# Improved Bioactivity of Titanium-Based Surfaces Fabricated by Laser Melting Deposition by Functionalization with 3D Polymeric Microstructures Produced by Laser Direct Writing via Two-Photon Polymerization

**DOI:** 10.3390/polym17192620

**Published:** 2025-09-27

**Authors:** Bogdan Stefanita Calin, Roxana Cristina Popescu, Roxana Gabriela Ghita, Eugenia Tanasa, Sabin Mihai, Irina Alexandra Paun

**Affiliations:** 1 Center for Advanced Laser Technologies (CETAL), National Institute for Lasers, Plasma and Radiation Physics, RO-077125 Magurele-Ilfov, Romania; 2 Horia Hulubei National Institute for Physics and Nuclear Engineering IFIN-HH, RO-077125 Magurele-Ilfov, Romania; 3 Faculty of Medical Engineering, National University of Science and Technology Politehnica Bucharest, RO 060042 Bucharest, Romania; 4 Faculty of Applied Sciences, National University of Science and Technology Politehnica Bucharest, RO-060042 Bucharest, Romania

**Keywords:** laser melting deposition, laser direct writing via two photon polymerization, 3D microstructures, osteoblast-like cells

## Abstract

Titanium (Ti)-based implants are widely used for bone injuries but suffer from poor bioactivity. To address this, we propose an innovative synergistic approach that combines laser melting deposition (LMD) for the fabrication of titanium-based supports with laser direct writing via two-photon polymerization (LDW via TPP) for their functionalization with 3D polymeric microstructures. We functionalized Ti surfaces fabricated by LMD using Ti (99.85 wt.%) and TiC powders (79.95 wt.% Ti, 20.05 wt.% C), with 3D microstructures obtained by LDW via TPP. The 3D microstructures were made of IP-Dip photopolymer and comprised 64 vertical microtubes arranged in five layers (10 to 170 μm tall, >94% porosity). When seeded with MG-63 osteoblast-like cells, the Ti-based surfaces functionalized with 3D polymeric microstructures promoted 3D cells’ spatial organization. Moreover, the cells seeded on functionalized Ti-based surfaces showed earlier organic matrix synthesis (day 7 vs. day 14) and mineralization (higher deposits of calcium and phosphorus, starting from day 7), as compared with the cells from non-functionalized Ti. In addition, the traction forces exerted by the cells on the 3D microstructures, determined using FEBio Studio software, were of the order of hundreds of µN, whereas if the cells would have been seeded on extracellular matrix-like materials, the traction forces would have been of only few nN. These results point towards the major role played by 3D polymeric microarchitectures in the interaction between osteoblast-like cells and Ti-based surfaces. Overall, the functionalization of Ti-based constructs fabricated by LMD with 3D polymeric microstructures made by LDW via TPP significantly improved Ti bioactivity.

## 1. Introduction

Bone provides structural support, organ protection, and acts as a reservoir for minerals and biomolecules [1,2,3,4,5,6]. Its complex, multiscale architecture includes hierarchically organized nanostructures, such as hydroxyapatite, collagen fibrils, and water [1,2,3,4,5].

For many years, bone damage has been managed using metallic implants to sustain bone tissue during its healing process [2], but a major challenge of these devices was to improve their osteointegration, to facilitate its integration in the natural bone, resulting in its stabilization [2,3,4]. This process implies the migration of osteoblast cells at the implant surface, their adherence, differentiation, and mineralization [5].

Ballard et al. [7] examined the application of 3D printing technologies in medical practice and demonstrated that their use contributes to a reduction in operating room expenditures, as well as a decrease in procedure durations, particularly within the fields of orthopedic and maxillofacial surgery. Moreover, the incorporation of 3D-printed components into patient care has been recognized as a significant contributor to the overall value of healthcare systems. Signor F. et al. [8] investigated the application of molten salts as protective media during the debinding and sintering of Ti and TiNb alloys produced by metal injection molding (MIM) feedstocks. The approach aims to mitigate oxidation and contamination by creating an inert or controlled chemical environment, thereby improving the densification behavior, microstructural integrity, and mechanical performance of the titanium alloy. Recently, Chang C. et al. [9] fabricated high-performance Mg-RE alloy components using selective laser melting (SLM) and subsequently evaluated them, demonstrating favorable processing stability and mechanical performance. The authors systematically examine a representative WE43 alloy through the application of advanced material characterization techniques. The selected laser output was operated in the transition mode, enabling the production of nearly full-density samples (porosity = 0.85 ± 0.021%). The results confirm the considerable application potential of SLM-processed Mg-RE alloys and provide a foundation for their further development in biomedical engineering. Heinl et al. [10] fabricated novel cellular Ti6Al4V structures using the electron beam melting (EBM) technique for potential orthopedic applications. Micro-computed tomography analysis demonstrated the capability of EBM to produce 3D architectures with interconnected porosity, thereby facilitating vascularization and promoting appropriate tissue in-growth.

Currently, titanium (Ti) alloys are used on a broad scale in orthopedic applications due to their superior physical and mechanical properties [11,12]. Ti alloys are usually obtained through a solidification process/casting [13] or through powder metallurgy [14]. Generally, these conventional technologies imply many processing steps, which are time, material, and energy consuming. Additionally, the high reactivity of Ti with oxygen and its high melting temperature represents important challenges during the classical acquirement process of these alloys. The complexity of the extraction process, the difficulty at melting and the fabrication shortcomings increase the production costs because special machines are required [11,12,13].

Additive manufacturing [15,16,17,18] has shown significant potential in the orthopedic field, by facilitating the production of customized implants with complex geometries, adapted to the patient needs. Its advantages include reducing the production time, improving precision, as well as enabling the fabrication of porous structures that promote osteointegration [19,20,21].

In the last decade, the laser-assisted fabrication of Ti and Ti alloys emerged as a truly efficient approach to produce Ti-constructs for bone repair [14,22,23]. In particular, laser melting deposition (LMD) is an advanced technology for additive manufacturing, which enables the production of Ti and Ti alloys components directly with the desired shape, without any mold [24]. The LMD method has the capacity to produce almost completely dense components, starting from a computerized 3D model and offers many advantages, such as the capacity to produce complex forms, a high utilization of material, and low post-processing [25]. Thus, LMD is an attractive alternative to produce in situ Ti components, as well as composite materials with metallic matrix such as Ti and TiC [1].

It is known that optimal bone implants should mimic the specific morphological characteristics of the bone tissue, such as its spongy-like structure, formed by interconnected pores, with a significant surface-to-volume ratio, which favors the interactions between the tissue and the implant [1]. The main problem here is that these morphological characteristics are hard, if not rather impossible, to obtain in conventional metallic implants [1]. In particular, despite the advantages of conventional metallic implants made by Ti and alloys, it is difficult to obtain typical porous structures specific to trabecular bone, which drastically reduce their bioactivity [14,24]. To overcome their bioinert behavior, the surfaces of Ti-based implants have been topographically functionalized through various methods, the most salient of which being based on etching and coating [12,24,26].

In recent years, laser-assisted technologies have been employed to obtain different biocompatible layers, generally made of polymer materials, which improve the bioactivity of Ti-based implants [26]. One of the most efficient techniques to fabricate complex 3D architectures, including porous structures with controlled geometry and reproducibility at micro- and nanometric scale, is laser direct writing via two-photon polymerization (LDW via TPP) [27,28]. LDW via TPP is an additive manufacturing technique, a kind of 3D printing that allows the fabrication of 3D micro- and nanostructures with fully reproducible, complex architectures and having a spatial lateral resolution of about 100 nm [28]. In comparison to other additive manufacturing techniques, such as selective laser sintering, LDW via TPP employs the phenomenon of polymerization with two photons to create 3D polymeric microstructures with practically no constraints regarding the geometry [28,29,30,31].

This study introduces, for the first time, a synergistic combination of LMD and LDW via TPP, whereby LMD is employed for the fabrication of titanium-based supports and LDW via TPP is used for their functionalization with 3D polymeric microstructures. We, therefore, demonstrate an innovative approach to combine laser-assisted additive manufacturing technologies to improve the bioactivity of Ti-based surfaces for bone regeneration applications. Specifically, we functionalized Ti-based structures fabricated by LMD with 3D polymeric microstructures made by LDW via TPP, for accelerating the formation of mineralized bone matrix, in comparison with non-modified Ti-based surfaces. The novelty of this study not only comes from the architecture design of the 3D polymeric microstructures obtained using LDW via TPP, but also from investigating the mechanical interaction between the osteoblast-like cells and the 3D microstructures. In all, this study demonstrates a proof-of-concept on how laser-assisted technologies can be efficiently integrated to improve the Ti-based implants fabricated by LMD through functionalization with 3D polymeric microstructures fabricated by LDW via TPP.

## 2. Materials and Methods

### 2.1. Powders for LMD Fabrication of Ti-Bases Substrates

The metallic materials used for this study were spherical Ti powder (wt. % 99.85 Ti) and polyhedral TiC powder (wt. % 79.95 Ti, wt. % 20.05 C), with particle diameters ranging from 45 to 150 μm and 45 to 106 μm, respectively. The two types of powders were purchased from a commercial supplier, Atlantic Equipment Engineers, Inc. (Upper Saddle River, NJ, USA). The substrate used for the experiments was a 10 mm thick pure Ti plate. The physical properties of the base material and the filler materials in the form of metallic powder are presented in Table 1.

Before the experiments, both types of powders were subjected to heat treatment at 60 °C for 4 h in an oven (UN55, Memmert GmbH + Co. KG, Büchenbach, Germany) to remove the moisture absorbed by the particles.

The grinding and polishing steps are presented in Table 2.

### 2.2. IP-Dip Photoresist

Another important aspect in the LDW via TPP fabrication process of micro/nanostructures is the choice of the appropriate material. In this study, we employed Ip-DIP, a photopolymer that is compatible with the LDW via TPP method and that ensures optimal performance in the prototyping process was chosen. IP-Dip is one of the most encountered photopolymers in laser-assisted prototyping [32], available exclusively to users of the Nanoscribe systems. Among the advantages of the IP-Dip photosensitive polymer is the high resolution, combined with ease of use, and optimization for LDW via TPP use [33]. According to the information provided by the manufacturer, IP-Dip is a photopolymer specially designed for Nanoscribe’s immersion laser lithography (DiLL) technology, functioning as both immersion material and photosensitive material. Due to its refractive index, which is suitable for the focusing optics in the Nanoscribe equipment, IP-Dip provides ideal focusing and, implicitly, the highest resolution for this system. At the same time, this product is suitable for biomedical applications, being a biocompatible material according to ISO 10993-5, with no cytotoxic effects on living cells [34]. IP-Dip has a refractive index of 1.52 at a wavelength of 1.55 µm, which makes it ideal for advanced lithography technologies. It does not require pre-heating and uses a drop-casting process, thus simplifying handling. Exposure is performed at 780 nm for LDW via TPP. IP-Dip also does not require post-heating, the developing process being achieved using PGMEA/IPA immersion, providing an efficient and precise solution for creating 3D porous microstructures [33]. The material’s characteristics also include enabling lateral dimensions of the resulting structures of 150 nm, a low proximity effect, low stress, minimal shrinkage, and good adhesion to glass substrates. Information regarding the physical and mechanical characteristics of the IP-Dip photoresist [35] is presented in Table 3.

### 2.3. LMD Fabrication of Ti-Based Substrates

The experimental setup used for the fabrication of Ti-based metallic composite substrates (TMCs) is illustrated in Figure 2a. It consists of an integrated system, including a Yb:YAG laser source (TruDisk 3001, Trumpf, Ditzingen, Germany) delivering 1100 W of 1030 nm laser radiation in continuous mode and a robotic arm with 6 degrees of freedom (KR30HA, Kuka, Augsburg, Germany) on which a processing optics unit (BEO D70, Trumpf, Ditzingen, Germany) is mounted. The optics unit includes a powder delivery nozzle with 3 channels (Multijet nozzle SO16, Trumpf, Ditzingen, Germany) for powder guidance and a powder distributor (PF 2/2, GTV Verschleißschutz GmbH, Luckenbach, Germany). The powder is transported from the distributor via a hose (Ø6 mm) to the processing optics where, through the delivery nozzle, it is directed into the laser-focused spot on a substrate (Ø800 μm), with a “top-hat” energy distribution.

**Figure 1:**
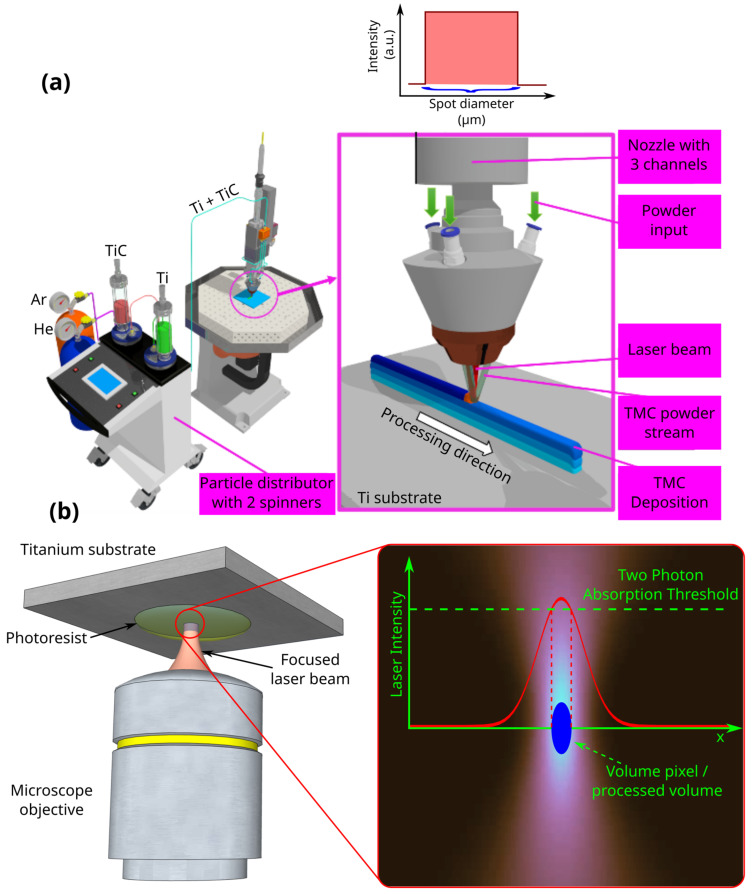
Schematic representations of (**a**) laser melting deposition (LMD); (**b**) laser direct writing via two-photon polymerization (LDW via TPP).

TMCs were obtained using the LMD processing technique, by the in situ mixing of the two components, Ti and TiC. Using a powder distributor equipped with storage cylinders, pure Ti powder, which constitutes the metallic matrix, was introduced into one container, while TiC powder, which represents the dispersed phase in the form of reinforcing particles, was placed in the second container. To select the desired TiC concentrations, the powder delivery rate was calibrated per minute for both Ti and TiC. For one revolution per minute (rpm), the titanium powder delivery rate was 1.926 g/min, while for TiC it was 0.697 g/min. The two types of powders were delivered simultaneously through hoses and directed into the laser beam via a nozzle, with predetermined flow rates. The laser beam melted both powders and formed the composite in its liquid phase, which rapidly solidified after the laser action ceased. To obtain the TMC base materials, Ti mixtures with a TiC content of wt.%1 were achieved by using 3 rpm for Ti and 0.1 rpm for TiC, which corresponded to the targeted concentration. Before the experiments, both types of powders were subjected to heat treatment at 60 °C for 4 h in an oven to remove the moisture absorbed by the particles. The LMD experiments were carried out using the processing parameters optimized in a previous study, namely a laser power of 700 W, processing speed of 0.01 m/s, and gas mixture of He (3 L/min)–Ar (10 L/min). To fabricate the substrates, it was necessary to optimize the scanning strategy to determine the spacing between the hatch lines (Δh) and the layer spacing in the vertical direction (ΔZ). The final optimal separation values were 0.5 mm for ΔZ and 0.25 mm for Δh. The scanning strategy required for the additive manufacturing process via LMD was generated using the computer-aided manufacturing program, TruTops Cell (Trumpf, Ditzingen, Germany). Substrates with dimensions of 16 × 16 × 2 mm^3^ were obtained (Figure 1a).

### 2.4. Laser Direct Writing via Two-Photon Polymerization (LDW via TPP)

**Figure 2:**
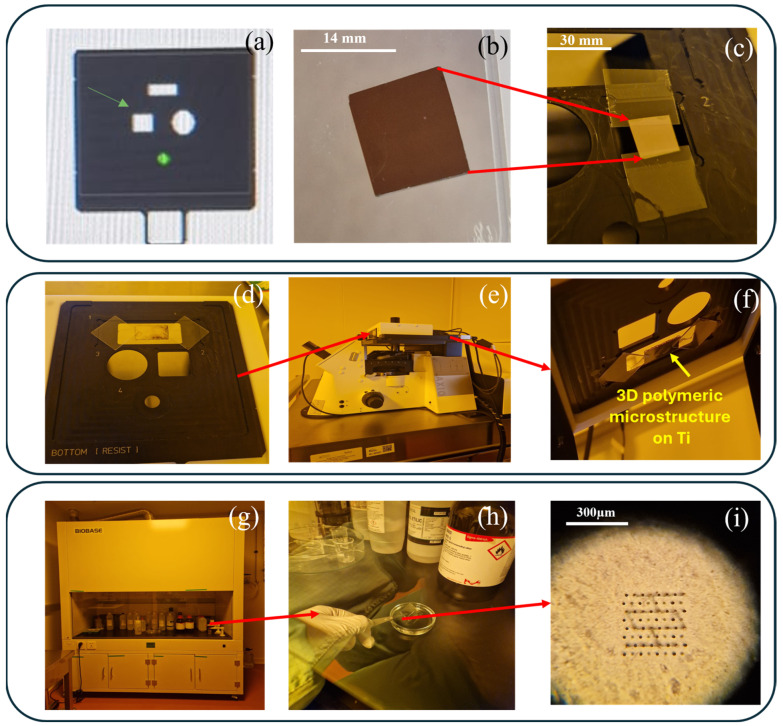
Experimental procedure for functionalization of Ti-based surfaces fabricated by LMD with 3D polymeric microstructures fabricated by LDW via TPP. (**Upper panel**) (**a**) Selected slot on Nanoscribe stand; (**b**) Ti-based substrate fabricated by LMD; (**c**) assembly of Ti substrate on the stand. (**Middle panel**) (**d**) Drop of IP-Dip photopolymer pipetted on Ti; (**e**) slot placed in Nanoscribe system (**f**) assembly after LDW via TPP fabrication of 3D microstructure on Ti-based surfaces. (**Lower panel**) (**g**) Post-processing step inside chemical hood; (**h**) development of 3D microstructure by washing away the unpolymerized material in PGMA; (**i**) 3D polymeric microstructure of Ti-based surface, as visualized through with an optical microscope.

In order to functionalize Ti substrates, 3D structures were designed using the SketchUp CAD software and printed using the laser direct writing via two-photon polymerization (LDW Via TPP) technique using the 3D lithography system Nanoscribe. (Figure 2b) schematically illustrates the LDW Via TPP technique. The laser writing system used to fabricate the microstructures was Nanoscribe Photonic Professional. The incident laser fascicle was generated using a pulsed laser with 50 fs duration, at a frequency of 80 MHz and a mean power of a maximum 120 mW. The wavelength was centered at 780 nm (NIR). The main phenomena responsible for the laser–matter interaction was the absorption of two photons. The photopolymer was transparent at the central wavelength of the incident wave, and the polymerization was mediated by a photoinitiator with high absorption for the employed laser wavelength [35,37].

### 2.5. Experimental Setup for Functionalization of Ti-Base Substrates with 3D Polymeric Microstructures

To insert the Ti-based substrates fabricated by LMD into the Nanoscribe equipment, a suitable support was chosen. Due to the substrate’s small dimensions, some adjustments of the experimental setup were needed, as shown in Figure 2c. As such, we selected the most appropriate sample holder (Figure 2a), where the Ti-based substrate (Figure 2b) was placed and glued between two glass slides 170 µm thick. Then, IP-Dip was applied onto the Ti-based substrate (Figure 2d), and then the sample holder was inserted into the Nanoscribe system (Figure 2e,f). After laser writing of the microstructures, the remaining unpolymerized monomer was removed by immersing the sample in PGMA (propylene glycol monomethyl ether acetate) for 12 min, followed by a short immersion in a very volatile solvent, Novec, to avoid deformation of the polymer structures, in this way reducing the capillary tensions. Considering the high aspect ratio of the structures, the immersion and extraction of the samples from the developer were slowly performed, at an angle of approximately 45º, to avoid any mechanical deformation of the structures. The entire process took place inside the chemical hood, to avoid inhalation of potentially irritating vapors of the solvents and to avoid contamination of the clean room.

### 2.6. Morpho-Physical Characterization of Ti-Based Substrates

Upon completion of the deposition experiments, all samples underwent detailed metallographic preparation and microstructural characterization using both optical and scanning electron microscopy. Specimens were sectioned using a Brilliant 200 precision cutting machine (ATM, Mammelzen, Germany) operating at a disk rotation speed of 2850 rpm. Following sectioning, the samples were hot-mounted in bakelite using an Opal 410 mounting press (ATM, Mammelzen, Germany), under a pressure of 50 bar and a temperature of 200 °C, with a mold diameter of 30 mm. Polishing was carried out to a mirror-like finish using a Saphir 520 system (ATM, Mammelzen, Germany), ensuring the surfaces were suitable for microscopic examination.

To reveal the microstructural features, the polished specimens were etched with Kroll’s reagent for 45 s. Optical microscopy was conducted using a Leica DM2700M RL microscope (Wetzlar, Germany) equipped with LED illumination and objective lenses offering magnifications of 5×, 10×, 20×, and 50×, complemented by a Leica MC190 HD digital camera for image capture. For high-resolution analysis, scanning electron microscopy (SEM) was performed using a Quanta Inspect S system (Eindhoven, The Netherlands). Quantitative analysis of the microstructural constituents—including granular eutectic phases, primary phases, and unmelted TiC particles—was conducted through the image analysis of both optical and SEM micrographs.

### 2.7. Morpho-Structural Analysis of the 3D Microstructures Imprinted on the Ti-Baseed Surfaces

To structurally characterize the functionalized implant, scanning electron microscopy (SEM) and energy-dispersive X-ray spectroscopy (EDX) were used. SEM is a versatile technique used for the surface characterization of materials at micro and nanoscale levels, obtaining high-resolution images of the analyzed surfaces [38]. In this study, scanning electron microscopy (SEM) investigations of the morphology of 3D polymer microstructures were performed using an FEI Quanta Inspect F Scanning Electron Microscope (SEM) (Hillsboro, OR, USA). Before SEM analysis, the samples were coated with a layer of Au approximately 7 nm thick. Using the same system, the chemical composition of the samples was investigated energy-dispersive X-ray spectroscopy (EDX).

### 2.8. In Vitro Evaluation of the 3D Microstructure Printed on Ti Surfaces

Osteoblast-like MG-63 cells (Cytone, Heidelberg, Germany) were cultured in Dulbecco’s Modified Eagle Medium (DMEM, PAN-Biotech GmbH, Aidenbach, Germany, batch no. P04-04510) culture medium, supplemented with 10% fetal bovine serum and 1% Penicillin–Streptomycin, under standard temperature and humidified conditions (37 °C, 5% CO_2_, 90% humidity). The cells were used for a maximum of 10 passages after thawing the batch purchased from the manufacturer.

#### 2.8.1. Cell Seeding

The 3D microstructures printed on Ti surfaces were sterilized by exposure to UV for 4 h and then immersed in culture medium, in order to extract any toxic residues that remained following the direct laser writing process. The sterilized samples were placed in 35 mm Petri-dishes and seeded with cells. The cells were cultured at a concentration of 30,000 cells/50 µL in complete culture medium, the pipetting being performed directly on the area with microstructures. The cells were then incubated for 40 min under standard conditions to allow their attachment to the structures, after which 5 mL of complete culture medium was added. The samples were then incubated under standard conditions of temperature and humidity for periods ranging from 24 h to 14 days.

#### 2.8.2. Preparation of Samples for Morpho-Structural Analysis

In order to analyze the morphology of the cells and their attachment to the 3D polymeric microstructures, SEM was employed. For this analysis, the cells were fixed, dehydrated, and subsequently covered with a 7 nm layer of Au. Following incubation, the cells were gently washed with PBS, then fixed for 1 h in 2.5% glutaraldehyde in PBS. Then, the cells were washed and incubated with different ethanol solutions with increasing concentrations (70, 90, 100%), for 30 min each. After these steps, the cells were incubated in solutions of hexamethyl disilazane HMDS: ethanol of different concentrations (50:50, 75:25, and 100:0, respectively), for 6 min each. Finally, the cells were completely dried and coated with a Au layer for visualization by SEM.

### 2.9. Simulation of the Traction Forces Exercised by Cells Cultured onto the 3D Microstructures

Cell-generated forces, known as traction forces, can play an important role in many physiological processes, and the study of single-cell mechanics is necessary to understand these functions. Particularly, micro-scaled structures are very useful to detect these forces through the deformations induced by cells, providing useful information in testing the phenomenon at the single-cell level. Cell-generated forces can be transmitted through proteins in the extracellular matrix or directly from cell to cell. These types of forces have demonstrated an important role in wound healing, tissue morphogenesis, and regeneration. They can influence cellular processes that affect both normal, as well as pathological states [39]. To better understand the cellular behavior on the resulting 3D microstructures, in particular to evaluate the traction forces exerted by cells on the 3D polymeric microstructures, we performed finite element analysis simulations using the FEBio Studio simulation environment [40].

#### 2.9.1. Finite Element Analysis of the Cells Cultured onto the 3D Microstructures

Finite element analysis (FEA) is a numerical method for predicting the behavior of objects or materials, based on the finite point method (FEM), which is a mathematical method for solving partial differential equations that describe the physical behavior of continuous systems [41,42]. FEM involves the discretization of complex systems into a finite number of subdomains (elements), which are connected by nodes. FEA is the application of the FEM used to validate experimental results or to test the mechanical strength of structures and architectures.

#### 2.9.2. FEBio Studio

The simulation environment chosen for the numerical simulations was FEBio Studio version 2.9.0 (Weiss Biomechanics Lab, University of Utah and the “Musculoskeletal Biomechanics Laboratory”, Columbia University, New York, NY, USA), an open-source software, specifically designed for solving nonlinear finite element analyses in the field of biomechanics and biophysics [40]. FEBio (Finite Elements for Biomechanics) was designed to model objects or structures made of nonlinear, isotropic, anisotropic, and time-dependent biological materials. The boundary conditions provided by FEBio were specifically designed to model biological interactions. Because deformable models can be connected to rigid models through adapted methods (for example, they can have a completely rigid connection, they can slide without interpenetrating, two unequal surfaces can be connected, etc.), it is possible to set the degrees of freedom of the movements and study the interactions between them [40].

#### 2.9.3. Preparing the Geometry and Choosing the Materials for FEB Studio

FEBio Studio allows the import of geometries from various external sources and accepts a wide range of basic file types, including STL files, as in the case of this work. The first step was to create a new model and choose the type of analysis. Considering the purpose of these simulations, namely to calculate the cellular traction forces exerted on some elements of the 3D polymer microstructure, the simulation type chosen was “structural mechanics”, i.e., analysis from a structural point of view. Next, we imported the main geometry for this simulation. In order to more closely simulate the experimental reality, we chose to add the titanium substrate, so that the link between the substrate and the microstructure could also be simulated. The substrate was directly designed in FEBio studio.

The correlation of the materials with the two geometries can only be achieved after performing a volumetric tetrahedral remesh on them, especially in the case of the geometry imported from an STL file (which only has a surface made of sets of triangles, not volume). We performed this process for both objects. In both cases, we chose the TeTGen mesh method to generate the tetrahedral mesh. The size of the elements was chosen according to two different criteria in the two cases. In the case of the microtube, the value was chosen according to the average size of the “triangles” in the original set, and in the case of the substrate, it was chosen arbitrarily.

The materials available in the FEBio Studio were chosen to be closely correlated to the real properties of the structures, subsequently modifying the values of the constants. In the case of the titanium substrate, a “rigid body” type material was chosen, the study of its deformation not being part of the objective of this work. For the IP-dip polymer, a neo-Hookean type material was chosen, due to its nonlinear character under mechanical stress. For the titanium-based substrate, the value for density was 4.94·10^−12^ g/µm^3^ and the value for Young’s modulus was 5·10^8^ kPa, chosen in accordance with the manufacturer’s instructions. In the case of the IP-DIP polymer, the variables were chosen in accordance with the specialized literature, varying depending on the laser power used for polymerization: for density, it was 1.2 × 10^−12^ g/µm^3^; for Young’s modulus, it was 1.55 × 10^3^ kPa; and for Poisson’s ratio, it was 0.35 [36].

After assigning the specific material to each object in the assembly to be simulated, the initial conditions were set. These include attaching the vertical microtubes to the substrate and conditioning the objects’ degree of freedom in terms of the direction in which they can move.

## 3. Results and Discussions

### 3.1. Morphological Investigation of Ti-Based Substrates

**Figure 3:**
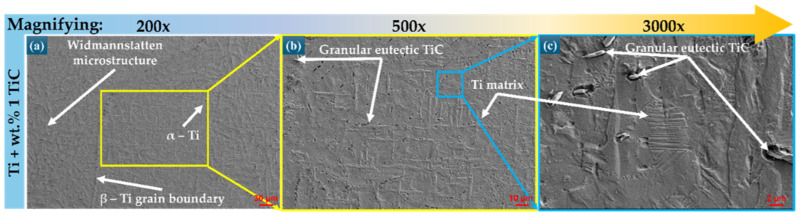
Electronic microscopy images of the composite materials microstructure with a content of wt.% 1 TiC in particle form, at different magnifications: (**a**) 200× (**b**) 500× and (**c**) 3000×.

Scanning electron micrographs of the TMC sample (Ti + wt.% 1 TiC) surface are presented in Figure 3, where dense structures free of defects such as pores or cracks can be observed. The Ti matrix exhibits an α + β microstructure, indicating that during the solidification of the metal, β phase grains were initially formed, which then resulted in the α phase. The crystal structure of pure Ti at ambient temperature and pressure is hexagonally close-packed (α), and after exceeding the temperature of ~890 °C, it undergoes an allotropic transformation into the body-centered cubic β phase, which remains stable up to the melting temperature. In Figure 3a, a dendritic α phase can be identified, consisting of elongated, light-colored grains, forming a “Widmanstätten” structure with a high degree of mechanical strength, typical of titanium and its alloys deposited by the LMD technique. TiC has a face-centered cubic (FCC) crystal structure, where Ti atoms form the face-centered cubic lattice with octahedral voids partially filled with C atoms, leaving the other spaces in the lattice vacant. However, the molar fraction of Carbon in TiC ranges from 0.32 to 0.49, and TiC can be treated as a solution with two sublattices, where Ti atoms completely occupy one sublattice and C atoms complete the other sublattice, forming a complete lattice [43].

### 3.2. Morpho-Structural Characterization of the 3D Microstructure Printed on Ti Surfaces

The first step in the LDW Via TPP fabrication process of the 3D microstructure printed on Ti surfaces was the creation of a 3D architecture. It consisted of microtubes of different heights positioned at distances of approximately 50 µm, aimed to stimulate bone tissue growth on structures with high porosity, comparable to the values reported in the literature as being similar to the spongy structure of natural bone [1,3,43]. This architecture aimed to guide the osteoblast-like cells towards the formation of a structure similar to the spongious bone. For this, the structure was made of vertical microtubes, placed on five levels, at different heights (the maximum height being 170 µm). The upper floors were anchored by a series of horizontal thin rods intended to maintain the vertical positioning of the microtubes. Thus, the architecture aimed to stimulate the detachment of cells from the 2D plane of the Ti-based surface and their growth in 3D geometries, developed vertically. In previous studies [44], we reported that osteoblast cells prefer smooth, rounded surfaces, which favor the cells anchorage and proliferation; therefore, a tubular architecture was chosen for the vertical pillars. In addition, for increased support, these pillars were not hollow inside but contained two additional cylindrical structures.

**Figure 4:**
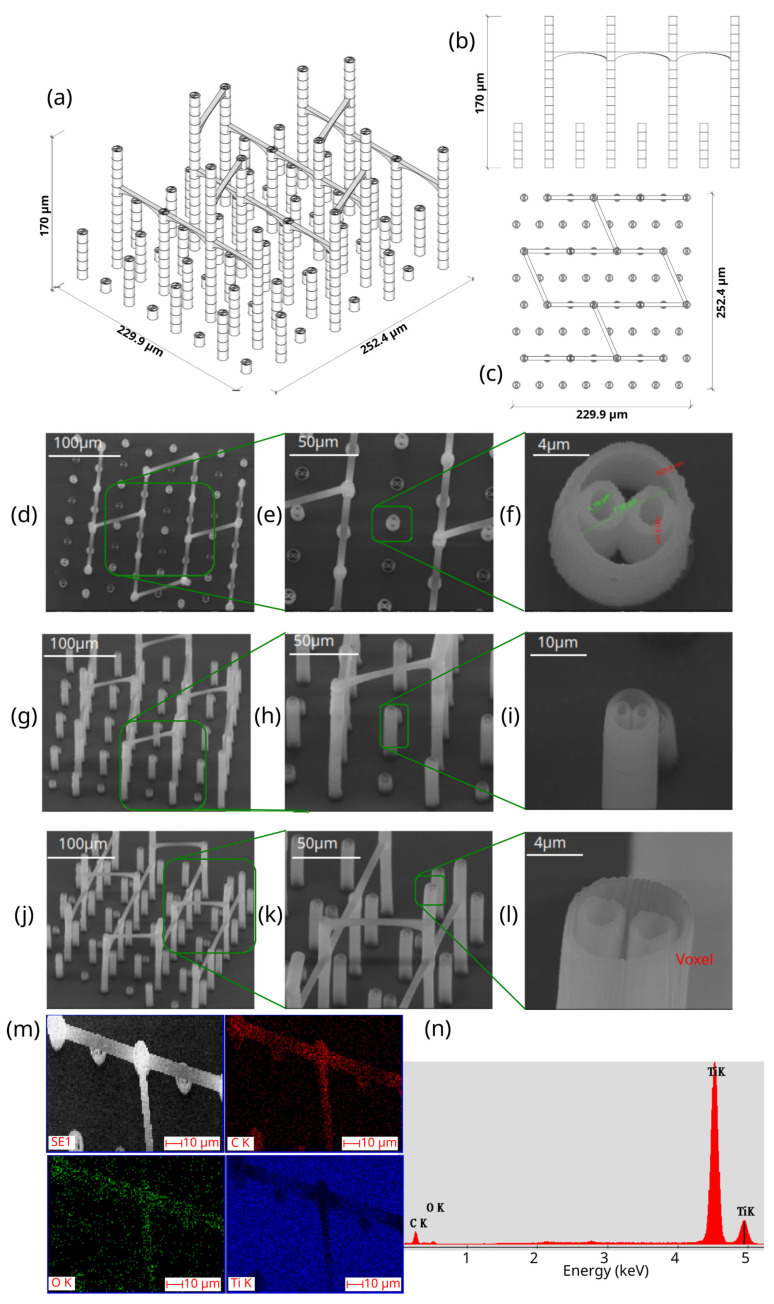
Design, fabrication, and structural characterization of 3D polymeric microstructures fabricated by LDW via TPP on Ti-based surfaces fabricated by LMD. (**Left panel**) Design of 3D microstructures: (**a**) overall view; (**b**) side view; and (**c**) top view. (**Middle panel**) Scanning electron microscopy (SEM) images of polymer structures made by LDW via TPP on Ti-based wafers fabricated by LMD: (**d**) top view of the assembly; (**e**) top view, detailed, of the microtubes in the 5 floors of the structure; (**f**) top view of a microtube; (**g**) overall view of the structure tilted by 20 degrees; (**h**) 20 degree tilted view of the microtubes on the 5 floors of the structure; (**i**) 20 degree tilted view of a microtube; (**j**) 40 degree tilted view of the assembly of the structure tilted by 40 degrees; (**k**) 40 degree tilted view of microtubes on the 5 floors of the structure; and (**l**) 40 degree tilted view of a microtube’s top. (**Right panel**): (**m**) EDX mapping and (**n**) EDX spectrum of 3D polymeric microstructures fabricated by LDW via TPP on Ti-based surfaces.

The detailed architecture is described in Figure 4a–c and Table 4 and Table 5. It contains 64 microtubes, equally distributed in eight rows and eight columns, with varying heights, in the range of 10–170 µm: the lower level has 10 µm, the middle level 50 µm, and the upper level 170 µm. The base of the structure is a rectangle with dimensions of 22.99 µm and 25.24 µm, respectively.

Figure 4 also illustrates the comparison between the designed (Figure 4 top panel) and fabricated structure imaged by scanning electron microscopy (Figure 4 middle panel). It can be observed that the architecture remained intact after the post-processing step despite the difficulties imposed by a large aspect ratio. Also, EXD investigations provided evidence that the 3D polymeric microstructures were printed on Ti-based substrates (Figure 4, lower panel).

The use of LDW Via TPP to functionalize Ti-based surfaces fabricated by LMD has offered several advantages, such as (1) the possibility to perform simple 3D processing, eliminating the need of masks or molds and allowing point by point precise 3D scanning; (2) the high resolution of this fabrication technology overcoming the optical diffraction limits, reaching 100 nm resolution, which ensures precise details of the processed structures; and (3) the low thermal effect during the processing, due to femtosecond laser pulses, which eliminate the conversion of the energy to heat, thus the material not being destroyed in the vicinity of the area where the photopolymerization takes place [28].

Porosity is defined as the fraction of empty space over the complete volume. In this case, the complete volume is defined by the orthogonal parallelepiped encompassing the structure for which porosity is calculated. All floors use the same length and width of the orthogonal parallelepiped that surrounds them, but different heights, depending on the height of pillars pertaining to each floor (see Table 4). Structure volume was determined using Blender 4.5.2 LTS with the 3D Print Toolbox. For example, for the complete structure:

- Total volume ~9,853,200 µm^3^
- Structure volume ~202,413 µm^3^
- Empty volume ~9,650,787 µm^3^
- Porosity ~97.9%

The porosity of the structure was calculated for all the floors overlapped, as well as for each floor separately (see Table 5). It should be noted that, in all cases, the porosity had values between 94 and 99%, being lower in case of the lower floors and increasing towards the upper floors of the structure. What is very important to note is that these porosity values are in fair agreement—at the upper limits—with previous studies referring to spongy bone tissue [45,46].

Despite the advantages of conventional metallic implants (mostly Ti and alloys), it is difficult to obtain typical porous structures like the trabecular bone [14,22]. It is known that optimal bone implants should mimic the specific morphological characteristics of the bone tissue, such as its spongy-like structure, formed by interconnected pores, with a significant surface-to-volume ratio, which favors the interactions between the tissue and the implant, but they are hard to obtain in conventional metallic implants [1]. To overcome their bioinert behavior, the surface of Ti-based implants has been topographically functionalized through various technologies, the most salient of which are based on etching, coating, and laser patterning. Etching is a more approachable method of stochastic surface functionalization that is still being studied to this day under specific conditions and has shown promising experimental results in various studies [47]. Surface functionalization via coating methods, while sometimes modifying the topography of the implant as well, generally aims to provide a biomimetic cell–implant interface, similar to the extracellular matrix of natural bone tissue.

In this context, our approach to functionalize flat Ti-based surfaces fabricated by LMD with 3D polymeric microstructures fabricated by LDW via TP allowed us to obtain fine tuning and full reproducibility of the imprinted microarchitectures; as such it can be very efficiently used to control the porosity of the Ti-based implants, with nanometer range resolution.

The morphological characteristics of the structure were analyzed by scanning electron microscopy (SEM). Figure 4d–l illustrates SEM images of the polymeric 3D microstructure printed on Ti surfaces by LDW Via TPP. The first important experimental observation is that the 3D microstructures were almost 100% compliant with the designed structure. Thus, in the overall model of the structure (Figure 4d–l), it is observed that the spatial arrangement of the microtubes coincided with the designed structure (Figure 4a–c). Additionally, the tilted SEM views (Figure 4g,h,j,k) show that all the other designed elements of the structure (vertical microtubes, arranged on five floors, supported by horizontal rods) were correctly and completely printed.

Another important aspect is that the structure printed by LDW via TPP maintained its morphological integrity after the developing process, despite the very high aspect ratio of the vertical microtubes (the ratio between the height of the vertical microtubes in the fifth floor of the structure and the diameter of a vertical microtube was 13.6) and, for this reason, presented a significant risk of tilting, or even collapsing, during the developing process of the structure in PGMA. Additionally, the five floors of the microstructure can be identified in the tilted SEM images (Figure 4j,k), each floor corresponding to a certain height of the vertical microtubes. Also, in these images, the horizontal micro-rods, which connect the microtubes of the five floors, can be identified, as well as their location at different heights on the structure. It is also important to mention that, although they have a large length in relation to their diameter (approximately 10 µm vs. 1 µm), these horizontal rods have preserved their structural integrity, as well as a few intersection points with the microtubes (approximately two points on lengths of approximately 40 µm).

In order to investigate with greater precision the degree of conformity between the structure printed with the LDW via TPP technique using the Nanoscribe equipment, with the designed structure, certain areas of interest were selected from the overall SEM images in Figure 4a,d,g, and Figure 4c,e,h. At a high level of magnification, the component substructures of the assembly were identified, namely the vertical microtubes and the horizontal support rods. The insets in Figure 4c,f,i show SEM images recorded at a very high magnification (20,000×) of the previously selected structural elements. In Figure 4f it was possible to measure, with sub-micrometric precision, the dimensions of the microtube walls as well as their diameter. Also, in these detailed images showcasing the interior of the hollow pillars, it can be seen that they contain two other non-concentric microtubes, located tangentially to each other, with a diameter of approximately ½ of that of the central pillar, having the role of a mechanical support. The detailed SEM images in Figure 4f,i,l also highlight the printing mode using the LDW via TPP technique, in which polymerization occurs exclusively in the photopolymer volume where the laser beam was focused, namely the voxel.

Next, in order to morphologically and compositionally characterize the 3D microstructures printed on Ti substrates, fabricated using LDW Via TPP from Ip-Dip photopolymer, EDX mapping analysis was performed. As can be seen in Figure 4m,n, the elemental mapping highlights the presence of titanium (Ti) on the base substrate; the presence of carbon (C), predominantly localized at the level of the polymeric microstructures; and the presence of a small amount of oxygen (O), that can come predominantly from the IP-DIP polymer, as well as from a possible oxidation on the surface of the titanium substrate. These results demonstrate the composition of the used substrate, but also the integrity of the structures fabricated on it.

Polymer–metal hybrid structures—such as those formed through joining, welding, or bonding—are increasingly utilized across a range of industrial sectors, including biomedical [48]. However, the substantial differences in the physical and chemical properties of polymers and metals present significant challenges to achieving strong and reliable interfacial bonding. Therefore, a clear understanding of the fundamental bonding mechanisms at the interface is crucial, as it provides the theoretical basis for further enhancing the performance of metal–polymer bonding.

The scientific interest in the fundamental properties of metal–polymer interfaces began in the late 1980s, as exemplified by early work such as that of P.S. Ho [49]. Since then, research focused on elucidating the interfacial bonding mechanisms between polymers and metals has grown steadily and continues to expand today [48,50].

To explore these interfaces, a variety of advanced characterization techniques have been employed. Scanning electron microscopy (SEM), transmission electron microscopy (TEM), and energy-dispersive X-ray spectroscopy (EDX) are commonly used to examine the morpho-structural features of metal–polymer interfaces [51].

In addition, hard X-ray photoelectron spectroscopy (HAXPES), particularly when combined with synchrotron radiation, has gained increasing attention due to its enhanced probing depth, making it highly suitable for analyzing buried interfaces [52]. Another powerful tool for investigating buried interfacial regions is time-of-flight secondary ion mass spectrometry (ToF-SIMS), which enables detailed chemical characterization at the nanoscale [53].

Regarding the aim of the present study, the stability and durability of the 3D polymeric microstructures on the titanium (Ti) substrate were found to be appropriate for the intended application. This conclusion is supported by the observation that the microstructures on Ti-based substrates retained their 3D architecture and morphological integrity—aside from minor bending attributed to traction forces exerted by osteoblast-like cells—following osteoblast-like cell seeding. Osteoblast-like cells are known for their strong adhesion and significant mechanical interaction with the substrate, even after just a few hours of culture. The fact that, after 2–3 weeks of continuous culture, no detachment of the microstructures from the titanium surface was observed, strongly indicating that the adhesion between the polymeric structures and the Ti substrate was sufficiently strong for the envisioned biomedical application. While a more precise, quantitative assessment of interfacial adhesion would certainly be beneficial, it lies beyond the scope of the current study and would be more appropriately addressed in a dedicated investigation focused on metal–polymer interface characterization.

The scope of our study was not to investigate the bioactivity of titanium-based surfaces per se, as this topic has already been extensively explored in the literature, with numerous studies published since the early years of biomaterials research [54]. Instead, our objective was to present a proof-of-concept for enhancing the bioactivity of titanium surfaces by coating them with 3D polymeric microstructures with controlled geometry. Therefore, laser direct writing via two-photon polymerization (LDW Via TPP) acts as the main focus of the present study, as opposed to being ancillary to the objective. Our investigation is centered on the design and fabrication of 3D polymeric microstructures by LDW via TPP, with the goal of improving the osteointegration of titanium-based substrates. Consequently, the topography of the underlying titanium substrate is also not the primary focus of our work, but it is described in Figure 3, as well as in the Results and Discussions Section: “the substrate consists of dense, defect-free structures—typical of the laser melting deposition method—without pores or cracks”. Our emphasis is placed on the topographical characteristics of the 3D polymeric microstructures fabricated on the titanium surface, presented in great detail in Figure 4d–l.

### 3.3. The In Vitro Biocompatibility Assessment of the 3D Microstructure Printed on Ti Surfaces

The in vitro cultivation of osteoblast-like cells is a convenient and interesting method for testing biological processes related to bone tissue formation. The MG-63 cell culture is a model that produces abundant collagen-rich extracellular matrix (ECM) [55], particularly useful because it facilitates the study of bone ECM mineralization in vitro, which is essential to understand the biology of bone tissue formation [55,56,57,58,59]. Recent studies [55,56,57,58,59] have reported that osteoblasts produce and assemble an abundant extracellular network of collagen fibrils, which progressively accumulates over the first 6–8 days of the culture. Osteoblasts continue to synthesize collagen but fail to externalize it and assemble the collagen network [55]. The ECM layers in culture are reported to be composed of densely packed bundles of collagen fibrils, with 64 nm periodicity of type I collagen fibers [57,58,59]. In areas of mineralization in the ECM, some collagen fibrils were covered with small globular masses with a diameter of approximately 100 nm [58,59]. Backscattered electron SEM imaging, where elements with higher atomic numbers appear brighter, together with X-ray microanalysis, indicated that these globular structures and adjacent mineralized collagen fibrils contained high levels of calcium and phosphorus [59]. Although often isolated, small globular masses appeared as dense high atomic number areas, representing large mineral deposits [58,59]. Mineralized collagen fibrils were also evident in most areas of mineralization, as well as in small mineral nodules (~150 nm in diameter) located between collagen fibrils [57,58,59]. These small nodules may correspond to the small globular masses observed by SEM, while larger areas of mineralization may correspond to aggregated globular masses [55].

**Figure 5:**
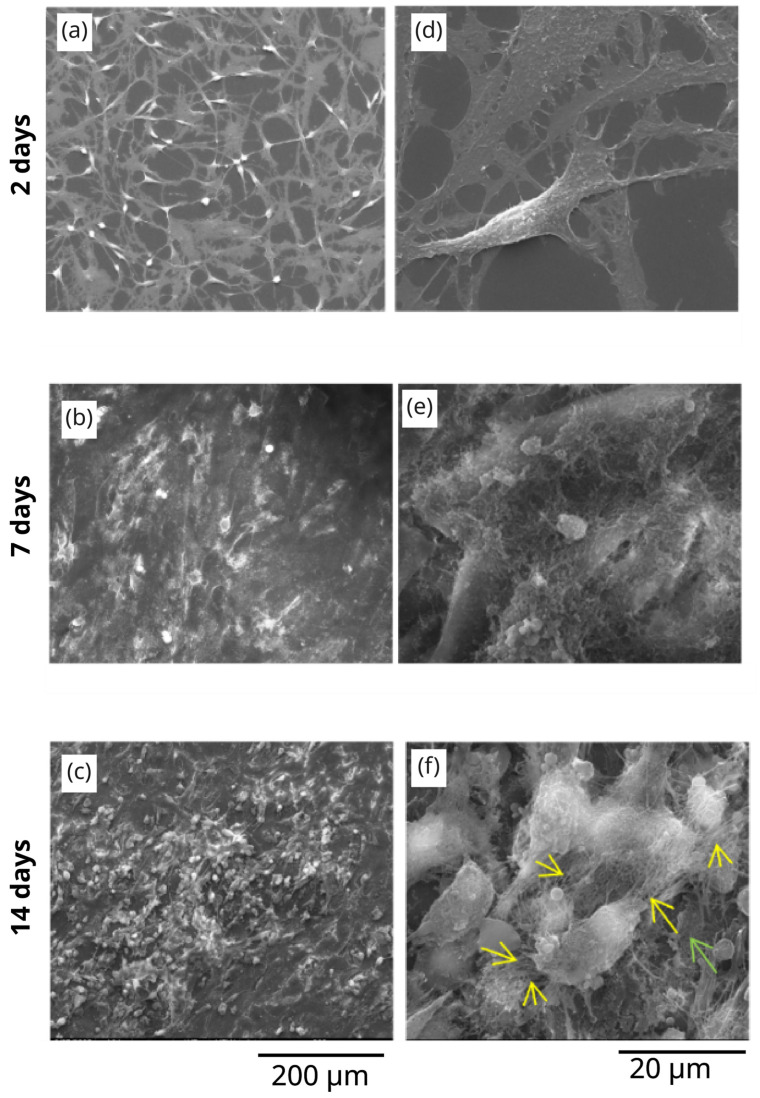
Scanning electron microscopy images of MG-63 osteoblast cells seeded on (**a**–**c**) flat titanium substrates (positive control); (**d**–**f**) detailed images of (**a**, **b,** and **c**, respectively). The images were recorded at different culture times, as indicated in the image.

**Figure 6:**
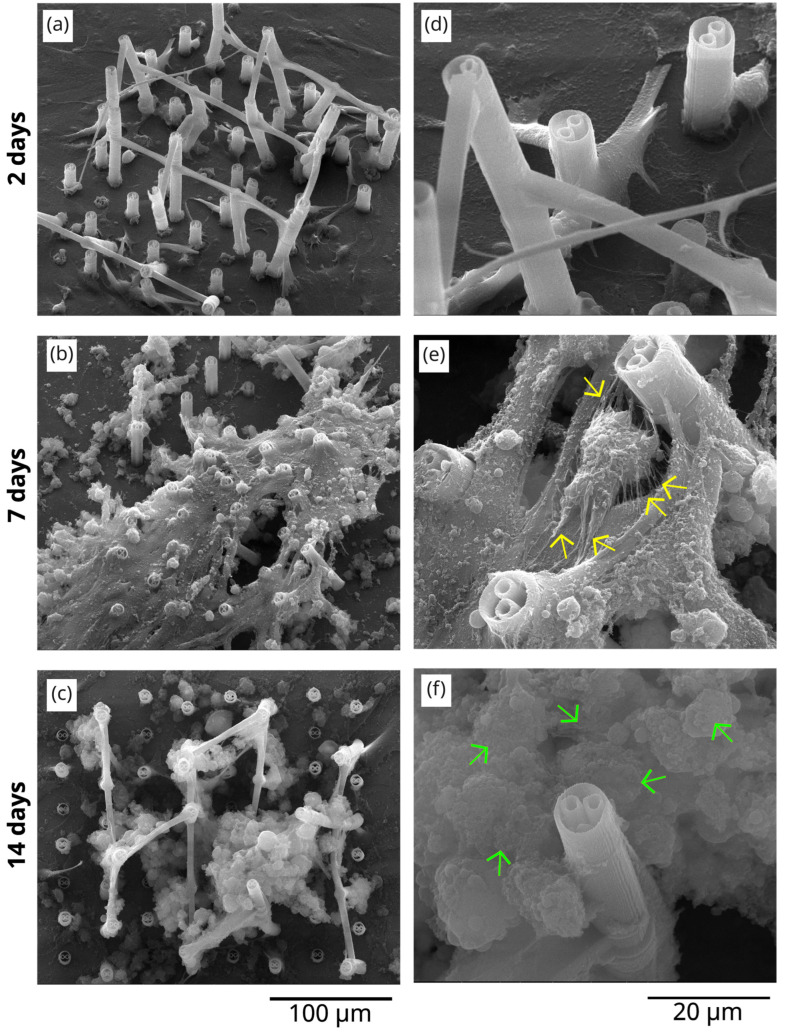
Scanning electron microscopy images of (**a**–**c**) Ti-based surfaces functionalized with 3D polymeric microstructures; (**d**–**f**) detailed images of (**a**, **b,** and **c**, respectively). The images were recorded at different culture times, as indicated in the image.

The results regarding the in vitro tests of our samples, carried out in osteoblast-like cell cultures, are presented in Figure 5 and Figure 6, which shows a detailed SEM analysis of the morphology of the cells seeded on flat Titanium-based substrates (Figure 5) and on 3D polymeric microstructures fabricated by LDW via TPP on Ti-based substrates (Figure 6). The analysis was performed at different culture times (2 days, 7 days, and 14 days). For a more correct and complete understanding of the experimental observations derived from these images, the obtained results were compared with the negative control samples, which are osteoblast-like cells seeded on Ti-based flat substrates, at the same culture times. Thus, in Figure 5a–f, images of MG-63 osteoblast cells seeded on flat titanium-based substrates (negative control) are illustrated.

In Figure 5a, corresponding to a culture time of 2 days, it can be observed that the cells are flattened on the 2D substrate and present a normal polygonal shape, with extensions. The detailed image at the level of a few cells in Figure 5d confirms these observations, which are in full agreement with the well-known characteristics of osteoblast-like cells, namely that these cells are strongly adherent [60]. Overall, it is observed that, on the Ti-based substrates, the cells are organized exclusively in a 2D assembly.

In contrast, for the same culture time of 2 days, the cells seeded on the 3D microstructures fabricated on the Ti-based substrate, in Figure 6a, start to climb on the microstructures, indicating an incipient tendency to become organized in 3D environments, according to the architecture of the structures. The cells, being strongly adherent, start to climb onto the microtubes of the structure and to attach to them, as illustrated in more detail in Figure 6b.

Upon increasing the culture time to 7 days, the cells reach confluence on titanium-based 2D substrates (Figure 5b), being completely interconnected, with no free space between them, reaching a confluence state. In the detailed view in Figure 5e, it is observed that the cells have been covered with a non-homogeneous and continuous layer of material. According to other published studies [55], this layer of material is most likely extracellular matrix that starts to be secreted by the cells.

An interesting behavior of the cells was observed in the case of cells seeded on 3D polymeric microstructures on titanium-based substrates, after 7 days of culture (Figure 6b). In this case, the cells seem to have climbed onto the upper part of the structure, partially populating the inferior floors. The cells seem to be maximally extended, in their attempt to attach to the neighboring microstructures. These extensions of the cells’ filopodia prompted their closer investigation. Thus, in the detailed image in Figure 6e, filaments of tens and hundreds of nm in diameter can be observed, some of them being marked with yellow arrows, for easier observation. These filaments are very clearly “expressed” in the case of all cells on the microstructure. According to relevant publications in the field [55,56,57], these could be assigned to organic matrix fibrils secreted by cells during their evolution towards mature bone tissue.

In parallel, for 14 days of culture, the cells on 3D microstructures show again a more advanced stage of maturation than the cells on flat substrates. More precisely, in Figure 6c, the 3D microstructures seem invaded by micrometric globular formations, which can be attributed to the formation of mineralization zones [55,56,57,58,59]. Figure 6f illustrates a detailed image of these formations, which are marked (for easier visualization) with green arrows.

**Figure 7:**
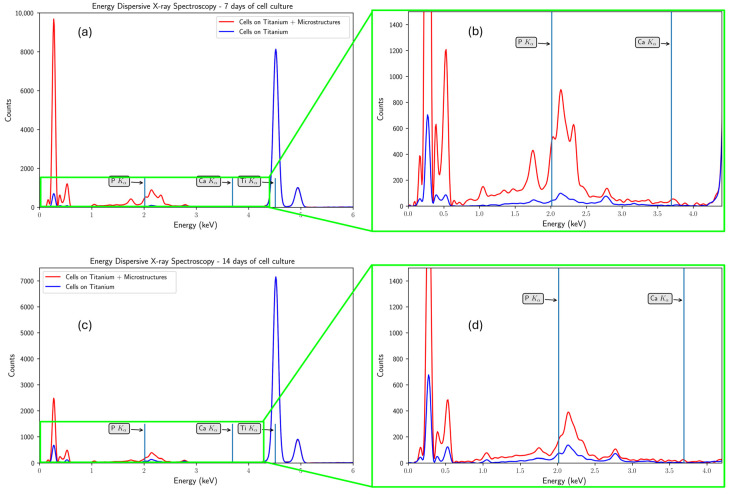
EDX spectrum after 7 and 14 days of culture. The red line corresponds to Ti functionalized with 3D microstructures and the blue line represents the tun-functionalized Ti: (**a**) 7 days of cell culture; (**b**) inset of interest region from (**a**); (**c**) 14 days of cell culture; (**d**) inset of interest region from (**c**).

To highlight the clear difference between cell differentiation on the titanium-based substrate functionalized with polymeric microstructures, compared with their proliferation on the Ti substrate alone, Figure 7 presents the spectra obtained using energy-dispersive X-ray microanalysis in comparison for the 2D and 3D culture conditions. In this figure, the higher phosphorus level on the 3D substrate can be observed (red line), compared with the non-functionalized one (blue line). However, in order to conclude on the effectiveness of the microstructures in producing faster osteointegration, more detailed experiments are needed, with a higher cell density in the culture performed and with longer culture times.

In our study, we employed the MG-63 cell line, which is a well-established osteoblast-like immortalized cell line [61] and is characterized by an accelerated metabolic activity. Consequently, the mineralization processes in a simulated environment involving MG-63 cell cultures occur at an increased rate compared to other biological models [62]. This enables us the detection of mineralization effects at shorter incubation times, such as 14 days, which streamlines the monitoring of this biological process. While longer-term experiments could yield to more insight into the stability of the mineral deposits, the use of MG-63 cells provides a rapid and reliable indication of the mineralization potential in the 3D environment.

These results are very well explained by previous studies on mineral deposits in the form of hydroxyapatite (HA). HA is a natural mineral of calcium phosphate, formed as platelet-shaped crystals (2–10 nm thickness, 20–50 nm length, and 15–30 nm width [63]), and found between collagen fibrils in bones. Both the collagen fibrils and the HA crystals are positioned in a highly ordered hierarchical system that offers specific mechanical properties, such as strength, elasticity, energy dispersion, and the stopping of crack propagation [63]. Bone mineralization is a highly complex biochemical process that results in the formation and deposition of HA crystals in specific locations between the collagen fibrils. While a consensus is not fully reached regarding HA crystal formation and propagation, more recent evidence supports the hypothesis that early mineralization begins in the matrix vesicles (MVs), which are organelles found on the membranes of osteoblast cells [64,65]. MVs accumulate Ca^2+^ and inorganic phosphate (IP), initiate the formation of the HA crystal, and subsequently break down, exposing the initial HA crystals to the extracellular environment, which further determines the propagation of the HA crystals and formation of the platelets between the collagen fibrils [65]. Apart from phosphorus-based enzymes, such as alkaline phosphatases, that play a key role in bone mineralization, inorganic phosphate, and inorganic pyrophosphate (IPP) can be considered some of the main components that ensure phosphate homeostasis in bones, i.e., IP has been shown to promote mineralization, while IPP is a strong mineralization inhibitor [65,66,67]. Plasma phosphate imbalance can indicate either a specific bone remodeling step (i.e., bone resorption results in higher phosphate plasma content, while bone mineralization results in a lower concentration) or a bone pathological condition such as rachitis/osteomalacia (also known as. “soft bones”, believed to be caused by an interruption in HA crystal propagation onto the ECM) [63,67]. However, during new bone formation, phosphate contents first drop in the first two weeks, as bone mineralization begins to occur, slowly increasing afterwards until reaching homeostasis [67].

Although there is no direct evidence on the long-term biodegradability of the IP-Dip acrylic-based photoresist, it is commonly used in high-resolution microfabrication applications for various implantable devices [68,69]. However, its properties can be modulated through polymer blending and crosslinking degree [70]. Despite its non-biodegradable nature, IP-Dip has demonstrated excellent long-term stability and biocompatibility in biomedical applications, with studies showing its suitability for durable, implantable devices such as nerve interfaces that maintain structural integrity and performance over extended periods of in vivo use [71].

In conclusion, the in vitro tests indicated that MG-63 osteoblast cells seeded on titanium-base surfaces functionalized with the 3D polymeric microstructures synthesized organic matrix fibrils and showed a faster mineralization tendency than cells seeded on 2D non-functionalized titanium-based surfaces.

### 3.4. Traction Forces Exercised by Cells onto 3D Microstructures Using Finite Element Analysis in FEBio Studio

The structure used during the simulations was based on a microtube extracted from the architecture used in the experimental part and a rectangle with much larger dimensions, representing the titanium-based metallic substrate. Both objects were subjected to a tetrahedral remeshing process, a process necessary to be able to attribute the specific material to each one. In the case of the microtube, a neo-hookean type material was chosen, suitable for nonlinear elastic materials, as is the case with the polymer used, while a rigid material type material was used for the titanium substrate. After introducing the material parameters and the configuration of the entire system, including the titanium-based substrate and the 3D polymer microstructures, as well as choosing the surface on which the forces will be applied, we performed the simulation to determine the degree of displacement of a microtube. Considering that the final goal was to determine the total forces necessary to reach the same degree of deformation—bending—of the microtube as that observed in the in vitro tests, the simulated time does not need to be taken into account.

**Figure 8:**
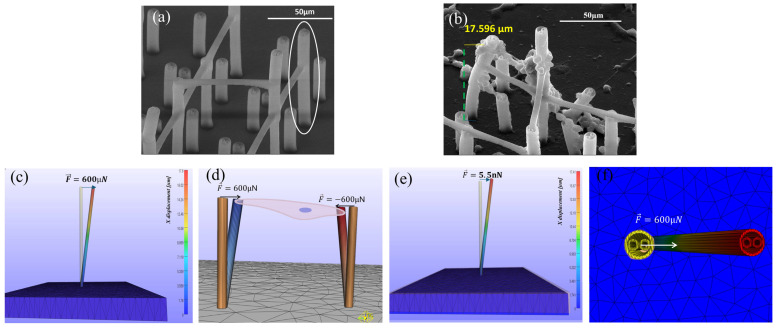
FEA simulation of the traction forces exerted by the seeded cells on the vertical microtubes form the 3D microstructures. (**a**) SEM image illustrating the initial state of a vertical microtube; (**b**) SEM image with vertical microtube from (**a**) bent, displaced cells seeded for 14 days; (**c**) FEA simulation of microtube’s displacement induced by the traction force exerted by cells from (**b**), to obtain a microtube displacement equal with the one measured form SEM image from (**b**) and considering Ip-Dip for materials’ parameters; (**d**) FEA simulation for two microtubes displaced under the action of traction forces exerted by cells attached on the tips of the microtubes, to obtain a microtube displacement equal with the one measured form SEM image from (**b**) and considering Ip-Dip for materials‘ parameters; (**e**) FEA simulation for the same case as in (**d**) but using materials parameters specific to extracellular matrix components. (**f**) Top View of FEA simulation for the case presented in (**c**).

The final goal of this simulation was to calculate the average traction forces exerted by MG-63 osteoblast cells on 3D polymeric microstructures fabricated by LDW via TPP on titanium-based substrates fabricated by LMD. In order to achieve this, a microtube was chosen which, according to the results of the in vitro tests, presented a visible displacement—bending due to a reduced number of cells. Following the study of the SEM images at all the culture times achieved, Figure 8b was chosen due to the clear visibility of a microtube that presents the previously mentioned characteristics. Another very important aspect for which this figure was chosen is the clear visibility of the lower surface of this microtube, which made it possible to calculate the displacement—bending using the Image J platform. Analyzing the figure more carefully, we could observe the reduced number of cells that affected the microtube. On its surface, we observed two main areas of cell adhesion, the most relevant one located on the upper surface of the microtube. Observing the orientation of the cells, we could clearly state that they are similar, thus affecting the displacement of the studied microtube approximately 1D, which facilitates a clearer understanding of the method of action of the traction forces. It is worth mentioning that SEM micrographs shown in Figure 8a,b are taken at a 40-degree angle with respect to the substrate normal direction, while FEA results are shown from a lateral perspective. Moreover, FEA takes into consideration only the elastic mechanisms of IP-Dip, while SEM micrographs show the results after 14 days of cell culture, where pillars have been subjected to culture media, various cell dynamics and repeated deformations.

In the case of the final simulation, due to the above-mentioned factors, we chose to apply a single force on the upper surface of the simulated microtube, its value being obtained according to the displacement—microtube bending; after processing, Figure 8b had an approximate value of 17.6 µm. The outer edges of the microtube were chosen as reference points for this calculation, as illustrated in Figure 8b. Initially, we illustrated the diameter of the base, according to which we drew a perpendicular line on it, tangent to the visible outer surface of the studied microtube. Finally, we drew a perpendicular to the tangent made starting from the same outer surface, this being the present bend.

Finally, having all the necessary data acquired, we performed the simulation of the traction forces acting on the fabricated 3D architectures. As can be seen in Figure 8c,f, the force applied to obtain a displacement similar to that in the in vitro experiments has a value of 600 µN. Also, in Figure 8c, the direction and sense of the applied force are represented by the blue arrow. The initial and final states, following the application of the force, are represented in this figure, the initial position being represented by the orange microtube, and the final one represented by the multicolor microtube, each color grade being assigned a bending calculated following the simulation. Also, on the right side of the image, the color map representing the displacement of the microtube compared with the initial position, expressed in µm, was shown; within the map, the maximum value was assigned to the upper surface, exactly as in the experimental case; this displacement has a value of 17.8 µm. Identical to the experimental case, the substrate remained motionless and the microtube remained anchored to the metal substrate, as can be seen by comparing the lower surface of the two states represented in this figure.

In the literature, the traction forces externed by cells on biological environments are generally in the order of nN [58]. However, most studies have reached this value by calculating tensile forces onto micro- and nanostructures made of very flexible materials, such as collagen or ECM, materials much less rigid than the IP-Dip polymer used to create the microstructures in this work [58,59]. Recent studies suggest that the generation of cellular forces is partly dictated by the elasticity of the surrounding matrix, where the cellular tensile forces increase with the stiffness of the substrate [56]. Therefore, to validate the results obtained in this work, we re-made the simulation with the same geometric characteristics, but using material parameters specific to the bone extracellular matrix. It has been shown that ECM-type environments (e.g., obtained in vitro from tunable polyacrylamide hydrogels coated with fibronectin) have a stiffness corresponding to Young’s modulus of 13–16 kPa [57]. For osteoblast-like cells, Poisson’s ratio for the ECM was estimated to be 0.5 [58]. Another material property necessary to determine the traction forces exerted by osteoblast cells on the components of the ECM is its density. The density of the components of the ECM is usually measured in milligrams per cubic millimeter (mg/mm^3^) [59]. In this simulation, we chose the following material properties for ECM [59]: an average density of 0.4495 mg/mm^3^, Young’s modulus of 15 kPa, and Poisson’s ratio of 0.4. In this case, a neo-Hookean material was also used to accurately simulate the nonlinear character of the ECM under mechanical stimulation. The simulation results on the microtube made of ECM are visible in Figure 8e, which contains the initial position of the microtube (represented in orange) and the final position obtained following the simulation, the maximum displacement being 17.41 µm. After performing this simulation, we observed that, in order to obtain a bending similar to the one reached in the in vitro tests, the applied traction force was 5.5 nN, which is in full agreement with the magnitudes of traction forces reported in the literature [59].

This result validates the correctness of the simulation performed for the 3D polymeric microstructures developed in this study. Thus, we can conclude that the traction force exerted by osteoblasts on the polymeric microstructures was three orders of magnitude higher than the forces reported in the literature for in vitro media, determined by the much higher rigidity of the polymer used to fabricate the 3D structures and compared with that of in vitro media (much more elastic media).

In perspective, these results provide the scientific background to develop 3D microstructures using LDW via TPP, with controlled porosity/architecture and specific material properties, which will enable the scientists to modulate the traction forces exerted by seeded cells and will further result in accurate control of the behavior of these cells in many important aspects related to the formation of functional tissue.

Further analysis of traction forces is required. SEM images presented in this work are taken after 14 days of cell culture, where IP-Dip structures have been repeatedly subjected to various cell movements and attachments that induce repeated deformations, both elastic and inelastic. FEA simulations, on the other hand, analyze a single adhesion point interaction between a cell and a pillar, i.e., a simplified approach (fewer variables and interactions) in order for the simulation to be better correlated with experiments. Therefore, complete stress–strain curve information [36] (i.e., inelastic deformations as well) should be taken into consideration in future calculations for IP-Dip structures, which also extends to multiple deformations that correlate to longer culture time, in order to obtain more accurate information.

## 4. Conclusions

In this study, we improved the bioactivity of titanium-based surfaces fabricated by laser melting deposition by functionalization with 3D polymeric microstructures produced by laser direct writing via two-photon polymerization. The porosity of the microstructures was calculated to be between 94% and 99% (depending on the microstructure layer), these values being in relatively good agreement with the values reported for the porosity of natural bone, although still being at the upper limit of these.

Based on the results of our study and within the limits of our knowledge regarding the current state of research in the subject addressed, we can state that the functionalization of titanium-based surfaces made by LMD with 3D microstructures fabricated by LDW via TPP favored the 3D organization of osteoblast-like cells and accelerated the synthesis of some extracellular matrix fibrils (which were evident after 7 days of culture), compared with cells seeded on non-functionalized titanium supports (where collagen fibrils were observed only after 14 days of culture). In addition, we found that the 3D polymeric microstructures accelerated the beginning of the mineralization process of osteoblast-like cells (incipient formations of Ca and P deposits, possibly related to the beginning of the mineralization process, being observed already after 14 days of culture), compared with non-functionalized titanium-based substrates, which did not present specific mineralization formations for the culture interval studied (14 days). Although in vitro studies with longer culture times are certainly needed, as well as structural analyses that would undoubtedly highlight the presence of calcium and phosphorus in cell cultures, as a sign of mineralization, the results obtained in this study indicate an improvement in osteogenesis in 3D cell cultures on 3D polymeric microstructures with porous architectures printed on Ti-based substrates.

Another interesting outcome of this study was obtained by simulating the traction forces exerted by the cells on the 3D microstructures. We found that the traction force exerted by osteoblasts on the polymeric microstructures was three orders of magnitude higher than the forces reported in the literature for in vitro media, the former being determined by the much higher rigidity of the polymer used to fabricate the 3D structures as compared with that of in vitro media (i.e., much more elastic media). In perspective, these results provide the scientific background to modulate the traction forces exerted by cells, seeded on 3D microstructures with well-defined architecture and material properties, which will enable accurate control over the behavior of these cells regarding important aspects related to the formation of functional tissue.

In all, these results offer promising premises for further studies regarding the use of lasers for the fabrication of Ti-based implants and their functionalization with 3D polymeric microstructures, which are expected to converge towards supporting patients in the process of osseointegration of bone implants and, consequently, improving their quality of life.

## Figures and Tables

**Table 1:** Physical–thermal properties of the commercial material used: Ti and TiC.

Crystal Structure	Hardness [HV]	Thermal Conductivity [W/m·K]	Melting Temperature [°C]	Density [kg/m^3^]	Material
α—HCP; β—BCC	117–202	17	1650–1670	4500	Ti
FCC	2500	21	3160	4940	TiC

**Table 2:** Grinding and polishing steps of TMC samples.

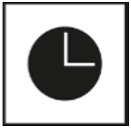 [min]	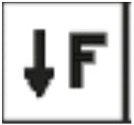 [N]	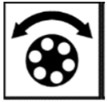	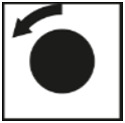 [rpm]	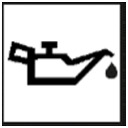	Medium	Step
Until plane	25	Synchronous rotation	250	H_2_O	SiC—paper P320	Planar grinding
2:00	25	Synchronous rotation	250	H_2_O	SiC—paper P600	Grinding
5:00	25	Synchronous rotation	120	Dia complete Poly, 9 µm	Beta	Pre-polishing
10:00 (H_2_O during final 0:30)	30	Counter rotation	120	Eposil F, 0.1 µm	Omega	Final polishing
0:45					Kroll’s reagent	Etching

**Table 3:** Physical and mechanical properties of IP-Dip material [35,36].

Refraction Index	Poisson Coefficient	Durity [MPa]	Young Modulus [GPa]	Density (Solid) [g/cm^3^]	Density (Liquid) [g/cm^3^]
1.52	0.35	152	1.55	1.2	1.14–1.19

**Table 4:** Dimensions of the component vertical microtubes in the 3D microstructures printed on Ti substrates, fabricated using LDW via TPP from Ip-Dip photopolymer.

5	4	3	2	1	Floor
170	130	90	50	10	Height [µm]
78.5	78.5	78.5	78.5	78.5	Surface [µm^2^]
17:1	13:1	9:1	5:1	1:1	Aspect ratio

**Table 5:** Porosity calculated for each floor of the microstructure and for the set of overlapping floors, respectively.

Porosity	Designed Structure	Floors	Porosity	Designed Structure	Floors
94.8%	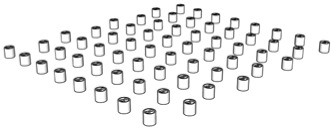	1st floor	94.8%	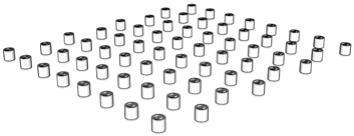	1st floor
96.2%	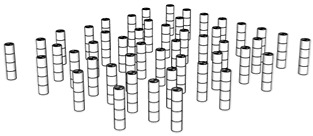	Only 2nd floor	95.9%	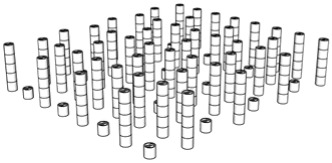	1st + 2nd floors
98.2%	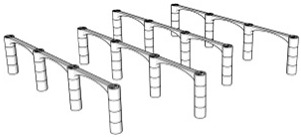	Only 3rd floor	96.9%	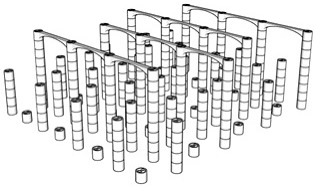	1st + 2nd + 3rd floors
98.7%	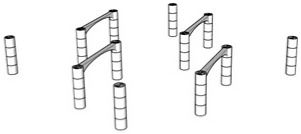	Only 4th floor	97.4%	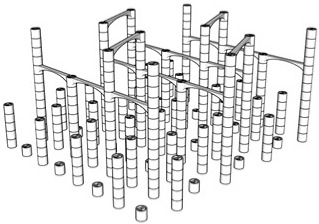	1st + 2nd + 3rd + 4th floors
99.6%	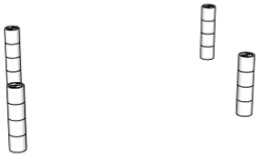	Only 5th floor	97.9%	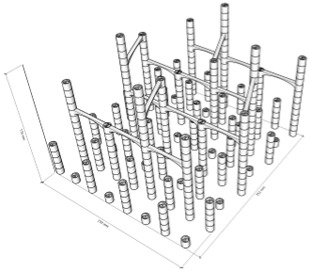	All floors

## Data Availability

The raw data supporting the conclusions of this article will be made available by the authors on request.
